# Response of Local Nitric Oxide Release to Manual Acupuncture and Electrical Heat in Humans: Effects of Reinforcement Methods

**DOI:** 10.1155/2017/4694238

**Published:** 2017-06-22

**Authors:** Sheng-Xing Ma, Paul C. Lee, Thomas L. Anderson, Xi-Yan Li, Isabelle Z. Jiang

**Affiliations:** ^1^Los Angeles Biomedical Research Institute at Harbor-UCLA Medical Center and Department of Obstetrics and Gynecology, David Geffen School of Medicine, University of California at Los Angeles, Torrance, CA 90502, USA; ^2^Los Angeles Biomedical Research Institute at Harbor-UCLA Medical Center and Department of Neurology, David Geffen School of Medicine, University of California at Los Angeles, Torrance, CA 90502, USA

## Abstract

This study was to examine the influences of manual acupuncture (MA) and electrical heat corresponding to reinforcing methods on nitric oxide (NO) release over the skin regions in humans. A device with collecting solution was taped to the skin surface along pericardium (PC) or lung (LU) meridian. Acupuncture needles were gently inserted into PC 4 with reinforcing stimulation (low force/rate) for 20 minutes in the MA group. LU11 on the finger was heated (43-44°C) by electrical heat for 20 minutes. Biocapture was consecutively conducted for two 20-minute intervals during and after each treatment. Total nitrite and nitrate (NO_*x*_^−^) in the collecting samples were quantified using chemiluminescence in blinded fashion. Baseline NO_*x*_^−^ levels are higher and tended to be higher over PC and LU acupoints during the 1st biocapture. NO_*x*_^−^ levels over PC regions were consistently increased by MA during both intervals. NO_*x*_^−^ concentrations over LU acupoints were increased and tended to be increased by electrical heat in the 1st and 2nd biocapture. The results suggest that reinforcing MA and electrical heat induce NO released from the local skin regions with higher levels at acupoints, which improve local circulation and contribute to the beneficial effects of the therapies.

## 1. Introduction

Manual acupuncture (MA) is traditionally used to treat many symptoms and diseases. Recent clinical trials reported reduced chronic pain associated with acupuncture and sham compared to nontreatment [1–3]. Interest in thermotherapy to treat diseases increased over the last few decades [4–6]. Several reports have demonstrated that acupuncture and heating cause multiple biological responses in both animals and humans [5–9]. These responses can occur locally at or close to the site of stimulation, or at a distance that is mediated mainly by the neuroendocrine system [1–3, 7–9]. However, the effects, biochemical changes, and mechanism responsible for the beneficial effects induced by the therapies are still unclear.

Acupuncture points (acupoints) are located along meridian lines (channels and collaterals, the jingluo) on the body surface, described in traditional Chinese medicine (TCM); and acupuncture comprises a family of procedures involving various techniques [10, 11]. Heat is considered as a reinforcement method and commonly used to treat a wide spectrum of syndromes associated with yang deficiency (sensation of coldness) [7–9, 11]. A reinforcement MA method involves slowly twisting/rotating the needle with gentle force/small amplitude [10–12]. Anatomical studies have identified that most acupoints are located intimately at the distribution of nerve fibers/trunks and blood vessels as well as the hair follicles and sweat glands are enhanced over acupoints [13–15]. Although acupuncture carries a long history, the biological effects behind the therapies are unclear. Particularly, the lack of tools and biomarkers to measure intervention outcome prevents building a solid biological foundation to study these interventions.

Nitric oxide (NO) plays a pivotal role in the generation of vascular relaxation and can be produced enzymatically by three nitric oxide synthases and nonenzymatically through the nitrate-nitrite-NO pathway in human skin [16–18]. Our previous studies demonstrated that NO synthase protein levels and NO contents are increased in skin tissue containing acupoints/meridian lines in rats [19–21]. Recent studies demonstrated that low-frequency electroacupuncture induces significant NO release following dermal microdialysis in the acupoint [22], and NO contributes to cutaneous vasodilation induced by acupuncture stimulation [23]. Repeated thermal therapy increases immunoreactivity and expression of endothelial NO synthase in the aortas of hamsters [24, 25], and NO level is increased in blood after warm needling in humans [26]. NO, with a half-life of a few seconds, rapidly oxidizes into nitrite and nitrate, and measurements of these stable metabolites adequately indicate changes in NO activity and production in tissues [27, 28]. Investigators have developed an NO-scavenging compound, 2-phenyl-4,4,5,5-tetramethylimidazoline-1-oxyl 3-oxide (PTIO), for use in biological systems [29, 30]. Our recent studies demonstrated, using a PTIO-infused biocapture device, that total nitrite and nitrate (NO_*x*_^−^) are increased over acupoints compared to meridian lines without acupoint (MWOP) and nonmeridian control region (NMCR) in humans [31–33], and this was later confirmed by another group using an alternative approach [34].

The purpose of this study is to determine the effects of MA and conductive heat on NO release using the biocapture method over the human skin surface of acupoints of the pericardium (PC) and lung (LU) meridians, compared to their corresponding MWOP and NMCR. Local NO release profile over the skin regions from MA with low stimulating rate/force was compared to conductive heat in humans.

## 2. Methods

### 2.1. Human Subjects

Twenty-five men and women (18–60 years old) recruited at Harbor-UCLA Medical Center volunteered for the studies (Table 1). Some subjects participated in more than one protocol in a randomized, blind, and noncrossover fashion. All subjects were healthy, normotensive, nonsmokers without major surgery in the past 12 months or history of cardiovascular disease. Subjects with dermatological problems, allergic diseases, vascular disorders, infectious diseases, and prescribed medication were excluded from study. The protocol was approved by the John F. Wolf, MD Human Subjects Committee of the Los Angeles Biomedical Research Institute at Harbor-UCLA Medical Center, and all experiments were performed in accordance with relevant guidelines and regulations. Informed consent was obtained from all subjects. Experiments were performed in a quiet, air-conditioned room with temperature maintained at 25–27°C.

### 2.2. Identification of Acupoints, Instrumentation, and Biocapture over Skin

**Figure 1:**
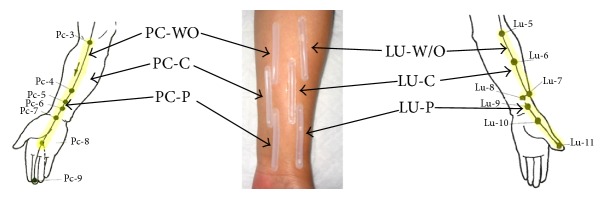
Representation of a biocapture device with a semicylindrical molded tube taped to the skin surface over meridians. The pericardium (PC) meridian and lung (LU) meridian lines and related acupuncture points are illustrated on left panel and right panel, respectively. The region from PC4 to PC6 is defined as acupoint (PC-P, 3 acupoints), the distance between PC3 and PC4 is defined as meridian line without acupoint (PC-WO), and nonmeridian control is the region close to PC meridian (PC-C). The region overlapping LU7 and LU8 is defined as acupoint (LU-P, 2 acupoints), the distance between LU6 and LU7 represents meridian line without acupoint (LU-WO), and nonmeridian control is nonmeridian area adjacent to the meridian (LU-C). LU11 denotes the acupoint in which a heat stimuli or moxibustion was applied, and a small probe sensor was placed on the surface of the heated area, and the temperature was controlled by a temperature controller. The device was adhered to the skin surface using a custom double-sided adhesive, and NO-scavenging solution (100 *μ*M PTIO) was injected into the tubing over the skin surface for 20 min in order to directly absorb NO.

Acupoints, MWOP, and NMCR over PC and LU meridians were studied in subjects as described in Figure 1. Locations were identified by an acupoint/meridian map of the human body [11, 35]. Regions were chosen based on consistency in identification and sufficient spacing for NO biocapture tube placement without contacting other meridians [31–33]. The biocapture method was described previously in humans [31–33]. A biocapture device, developed by this lab, consists of a semicircular molded plastic tube (0.5 × 5 cm) adhered to the acupoints, MWOP, and NMCR by a double-sided adhesive, as shown in Figure 1. PTIO solution (100 *μ*M) was injected inside the sterilized tube to absorb NO from the skin surface for 20 minutes [31, 32]. After incubation, the liquid was collected from the tubes. Following the 1st biocapture, the PTIO solution was replaced and collected after 20 min for the 2nd biocapture [33].

### 2.3. Manual Acupuncture (MA) and Electrical Heat

Disposable acupuncture needles (0.3 × 25 mm) were gently inserted at 5–8 mm depth at acupoint PC 4 (Ximen) in MA groups. PC4 was stimulated by delicately twisting the needles according to the standard reinforcement technique for 2 minutes or until a sensation of moderate “de qi” (feeling of soreness, numbness, distension, or pain) was achieved. The needles were manipulated for 2 minutes every 5 minutes for 20 minutes.

For electrical heating, a heating pad constructed from zigzag folding of a wire within fire-resistant cloth was connected to a temperature controller and 6–12-volt battery to generate conductive heat as described previously [36]. This pad was placed over LU11 (1 × 1 cm) and tied with an elastic band around the finger. A small sensor over the heated area was connected to a thermocouple thermometer and temperature controller (Harvard Bioscience Inc., Holliston, MA). The temperature was controlled at around 43-44°C by adjusting the distance between the skin and heat source.

### 2.4. Quantification of NO Metabolites

Total NO_*x*_^−^ concentration was measured in the biocapture solution using an ozone phase chemiluminescence method (NOA280i, GE Analytical Instruments, Boulder, CO) as described previously [31–33]. Briefly, samples are refluxed in the presence of 1.5 mM vanadium (III) chloride in 2 M HCl which quantitatively reduces both NO_2_^−^ and NO_3_^−^ to NO gas. This can then be quantified by chemiluminescence detection after reaction with ozone. The quantitative analysis was based on the standard curve established by measurements of peak areas of the standard NaNO_2_ compound. All samples were measured in duplicate. The lower limit of detection of this assay was 0.1 pmol NO.

### 2.5. Research Protocols

Volunteers were randomly asked to participate in either MA over PC meridian or heat treatment over LU meridian. A biocapture device was adhered to both ventral forearms along acupoints, MWOP, and NMCR, as shown in Figure 1. MA stimulation was performed at PC acupoints, and electrical heating was applied at LU acupoint on a randomly selected arm. Two consecutive biocaptures of NO_*X*_^−^ were performed during and after treatments for 20 minutes each, which include cumulative NO on the skin surface present in the 1st biocapture and de novo NO synthesis/release in the 2nd biocapture. The opposing arm served as control for two consecutive biocaptures at 20 min each as the baseline NO_*x*_^−^ concentration without treatment over corresponding PC or LU regions [22, 33].

### 2.6. Statistical Analysis

Results were expressed as mean ± standard error of the mean (SEM) of NO_*x*_^−^ concentrations over the skin surface measured in the biocapture solution (*μ*M) and efflux rate calculated over the surface area during 20 min (nmol/cm2) along the skin surface in contact with the solution, respectively. The significance of differences was determined by three-factor repeated Analysis of Variance (ANOVA), where the three factors are (1) time intervals, 1st and 2nd biocapture; (2) three sites, acupoints, MWOP, and NMCR; and (3) treated side and untreated side. *P* values less than 0.05 were considered significant.

## 3. Results

### 3.1. Baseline NO Releases over PC Regions

**Figure 2:**
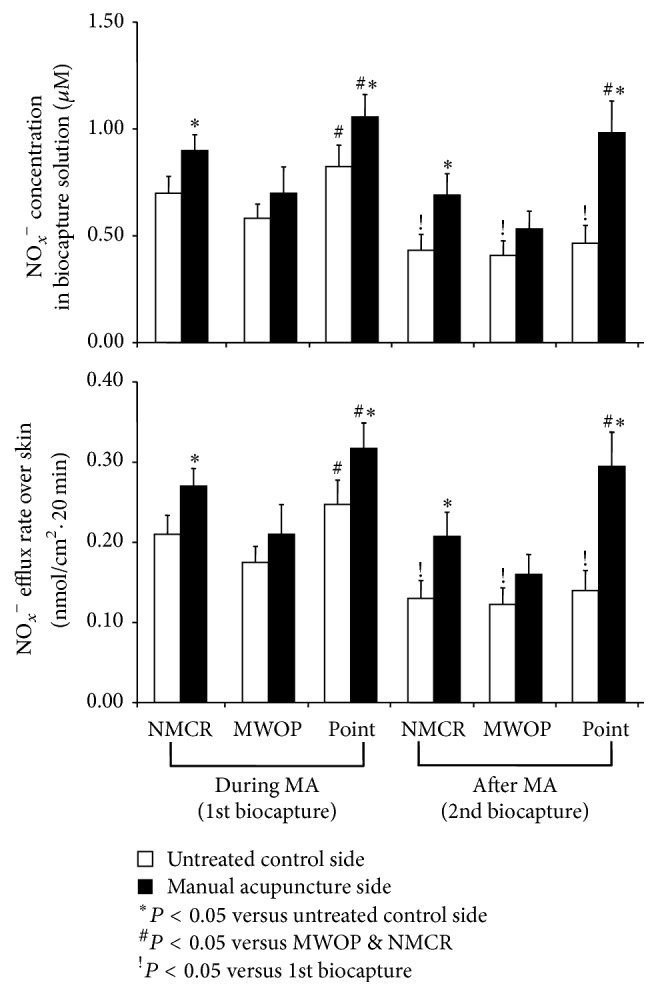
Quantification of NO metabolites over the pericardium meridian following manual acupuncture (MA). Concentrations of total nitrite plus nitrate (NO_*x*_^−^) along the PC meridian were collected over acupoints, meridian lines without acupoint (MWOP), and nonmeridian control region (NMCR) during 20 min of MA (the 1st biocapture, the left panel) and 20 min after MA (the 2nd biocapture, the right panel) in healthy volunteers. MA was performed on PC4 in one arm selected at random, while the opposing arm served as control. NO_*x*_^−^ concentrations in the biocapture solution (*μ*M, top) and efflux rate calculated over the surface area in contact with the biocapture solution over a 20 min duration (nmol/cm^2^·20 min, bottom) were significantly increased over PC acupoints and NMCR by MA of PC4 in the stimulated side compared to the side without stimulation. Each bar represents the mean values and vertical bars represent SEM; ^*∗*^*P* < 0.05, compared with untreated side; ^#^*P* < 0.05, compared with MWOP and NMCR; ^!^*P* < 0.05, compared with 1st biocapture.

Figure 1 is a representative example of the defined PC and LU acupoints, MWOP, and NMCR. A biocapture device was adhered to the skin surface over the PC regions (left panel) and LU regions (right panel). The characteristics of the participants are detailed in Table 1. Baseline NO_*x*_^−^ concentrations over PC regions were examined in 12 healthy volunteers for 2 consecutive biocaptures, 20 min each. A two-way ANOVA revealed that NO_*x*_^−^ concentrations biocaptured during the 1st interval over PC acupoints, MWOP, and NMCR were markedly and consistently higher than the 2nd biocapture (*P* < 0.05, Figure 2). There is a significant difference among PC acupoints, MWOP, and NMCR (*F*_8,48_ = 10.5, *P* < 0.01). NO_*x*_^−^ concentration over PC acupoints was higher than those over MWOP and NMCR in the 1st biocapture (*P* < 0.05).

### 3.2. Effects of MA on NO Releases over PC Regions

NO_*X*_^−^ concentrations following MA of PC4 were examined over PC regions in 12 healthy volunteers (Figure 2). ANOVA analysis of NO_*x*_^−^ concentrations biocaptured during the 1st interval over PC regions in response to MA suggests a significant elevation of NO metabolites compared to control (*F*_1,11_ = 14.7, *P* < 0.01). NO_*X*_^−^ concentration biocaptured during MA was increased over PC acupoints and NMCR compared to the control side (*P* < 0.01) (Figure 2, left).

Figure 2, right panels, shows NO_*X*_^−^ concentrations in the 2nd biocapture at 20 min after MA compared to baseline control over PC acupoints, MWOP, and NMCR. A two-way ANOVA revealed significant differences in NO_*x*_^−^ concentrations between MA and nonstimulated sides over PC regions (*F*_2,22_ = 4.0, *P* = 0.03). In the 2nd biocapture after MA, NO_*X*_^−^ concentration was markedly increased over PC acupoints compared to control (*P* < 0.01). NO_*X*_^−^ level over PC acupoints after MA during the 2nd biocapture almost achieved the level of the 1st biocapture. At 20 min after MA, NO_*X*_^−^ concentration was also significantly increased over NMCR compared to control (*P* < 0.05). NO_*X*_^−^ level over MWOP suggested an elevation after MA but did not attain statistical significance (Figure 2, right panels).

### 3.3. Effects of Electrical Heat on NO Releases over LU Regions

**Figure 3:**
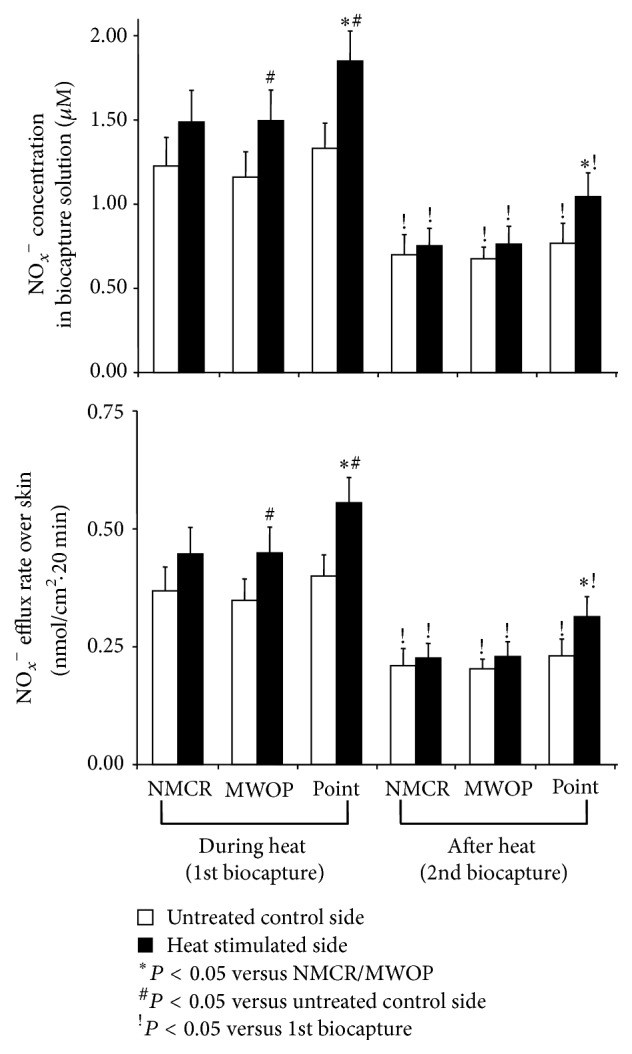
Quantification of NO metabolites over lung meridian following electrical heat. Concentrations of total nitrite plus nitrate (NO_*X*_^−^) along the lung (LU) meridian collected over acupoints, meridian lines without acupoint (MWOP), and nonmeridian control region (NMCR) during 20 min of electrical heating (the 1st biocapture, the left panel) and 20 min after heating (the 2nd biocapture, the right panel) in healthy volunteers. Electrical heat was performed on LU11 of one arm selected at random while the opposing arm served as control. NO_*X*_^−^ concentrations in the biocapture solution (*μ*M, top) and efflux rate calculated over the surface area in contact with the biocapture solution over a 20 min duration (nmol/cm^2^·20 min, bottom) over acupoints compared to MWOP and NMCR. Each bar represents the mean values and vertical bars represent SEM; ^#^*P* < 0.05, compared with unheated side; ^*∗*^*P* < 0.05, compared with MWOP and NMCR; ^!^*P* < 0.05, compared with 1st biocapture.

To determine the influence of electrical heat on NO releases, NO_*X*_^−^ concentrations were quantified over LU regions at 20 min during and after heating of LU11 in 13 healthy volunteers, as shown in Figure 3. There is not any subject feeling thermal pain or discomfort during electrical heating. NO_*x*_^−^ concentrations biocaptured during the 2nd biocapture over LU acupoints, MWOP, and NMCR were markedly reduced compared to the 1st biocapture (*P* < 0.05, Figure 3). Baseline NO_*x*_^−^ concentrations over LU acupoints tended to be higher than those over MWOP and NMCR in the 1st biocapture and the second 2 consecutive biocaptures, although these were not significant. Following heat stimulation, an overall significant difference was found by ANOVA analysis of skin regions among LU acupoints, MWOP and NMCR (*F*_2,24_ = 5.2, *P* = 0.03). NO_*X*_^−^ levels over LU acupoints on the stimulated side were significantly higher compared to the regions of MWOP and NMCR (*P* = 0.05). ANOVA analysis revealed an overall significant increase in NO_*X*_^−^ concentration following heat stimulation compared to control (*F*_1,12_ = 9.7, *P* < 0.01). NO_*X*_^−^ concentration was significantly increased over LU acupoints and MWOP during heat stimulation (*P* < 0.01). NO_*X*_^−^ concentrations biocaptured over NMCR during heat stimulation were moderately increased but did not reach statistical significance (*P* = 0.15).

During the 2nd biocapture after heat treatment, NO_*X*_^−^ concentration was marginally increased over LU acupoints compared to control (*P* = 0.11), as shown in Figure 3, right panel. NO_*X*_^−^ levels over LU MWOP and NMCR after heat treatment tended to be increased, but this difference fell short of statistical significance. After heat treatment, NO_*X*_^−^ concentration over LU acupoints was marginally higher than those over MWOP and NMCR (*P* = 0.08, *P* = 0.07, resp.).

## 4. Discussion

The purpose of this study was to examine two consecutive biocaptures of NO_*X*_^−^ over the skin surface of acupoints, MWOP, and NMCR along PC and LU meridians. The influences of MA with low stimulating rate/force on NO releases over the skin regions were compared to electrical heat in humans. The major new findings of these studies are as follows: (1) Baseline NO_*x*_^−^ levels are higher over PC acupoints than MWOP and NMCR during the 1st biocapture and markedly reduced over all PC and LU regions during the 2nd consecutive biocapture; (2) baseline NO_*x*_^−^ levels tended to be higher over LU acupoints than MWOP and NMCR during the 1st biocapture; the values are significantly higher following heating over LU acupoints compared to MWOP and NMCR; (3) NO_*X*_^−^ levels are moderately elevated during MA over the 1st biocapture and markedly increased after MA over the 2nd consecutive biocapture; and (4) NO_*x*_^−^ levels were increased over LU acupoints and MWOP regions with a higher elevating level over acupoints following heating during the 1st biocapture. This is the first evidence showing that NO is released/generated from the human skin surface with a higher level over acupoints by the reinforcing methods using electrical heat. MA with low rate/force-like reinforcement methods also increases local NO release over skin regions. NO contents biocaptured during the 1st interval, containing cumulative NO over PC and LU skin regions, are almost twofold higher than the subsequent biocapture, which mainly comprises newly generated NO. These results are consistent with our previous data, which reported that NO contents biocaptured during the 1st interval over PC regions are almost twofold higher than subsequent biocaptures, which suggest that both cumulative NO and newly generated NO exist on the skin surface [32]. NO contents during the subsequent biocaptures are reduced since the cumulative NO that mainly comes from nonenzymatic NO generation over skin surface was already removed by the 1st biocapture. The results demonstrate that cumulative NO and newly generated NO exist on the skin surface with higher levels over PC and LU acupoints especially following the treatments with reinforcement techniques. Enhanced NO over the 2nd biocapture at 20 min after ceasing treatments suggests de novo NO production-induced by the therapies, with a higher level over acupoints.

Based on TCM, reinforcement results in local feeling of warmness, but reduction causes local feeling of coldness. The findings presented suggest that NO levels biocaptured over the PC and LU acupoints are consistently increased by reinforcement methods, heating stimulation. MA by twisting/rotating the needle with gentle amplitude and moderate speed also elevates NO release predominantly over PC acupoints, which are similar to electrical heat. In contrast, NO levels over the ventral forearm skin regions are moderately reduced by the high frequency and force of EA stimulation (data not shown). The reinforcement methods induced elevations of vasodilator (NO) release over skin can increase local blood flow/microcirculation, which contribute to local warmness and their therapeutic effects. The precise mechanisms of the elevation of NO release affected by the stimulations applied are still unclear. A more sophisticated approach would be required to address this issue. Despite these limitations, our findings would be consistent with MA with low rate/force and electrical heat-induced elevations of NO release over PC and LU regions with a high level at acupoints and demonstrate a consistent response to NO release during reinforcement methods.

Recently, a large meta-analysis of patient-level acupuncture data for the treatment of chronic pain conditions has demonstrated that the effects of verum acupuncture on pain improvement have statistically significant, but small, differences compared to sham acupuncture procedures [1–3]. A number of acupuncture clinical trial projects mainly conducted by conventional scientists have generated many negative results. These have puzzled the acupuncture community, and published papers questioned whether the correct proposed acupuncture methods such as reinforcement and reduction were used in the trials [37]. Our results suggest that utilizing the appropriate parameters for an investigated technique and monitoring the quantitative response to the treatments are worth considering in acupuncture clinical trials. Moreover, the present data shows that cumulative NO is higher over acupoints at physiological level and stimulus-evoked NO release is also with a higher level at acupoints. Whether the effect of acupoint stimulation has better local effect than nonacupoint stimulation or how to select stimulating force/speed for specific diseases/symptoms requires investigation.

The mechanism responsible for an increase in NO release over acupoints by reinforcement methods is unclear. NO can be produced enzymatically by three NO synthases [16–18] and can also be produced nonenzymatically through the bacteria reduction pathway in human skin [31]. Present studies show that NO_*X*_^−^ concentrations over PC and LU acupoints are higher and tend to be higher than those in MWOP and NMCR in the physiological baseline level during the 1st biocapture but not in the 2nd biocapture. During MA and conductive heat treatments, NO_*X*_^−^ levels are elevated and higher over acupoints compared to their corresponding MWOP and NMCR during both biocaptures. Cutaneous vasodilation induced by acupuncture stimulation in the forearms of humans is attenuated by application of NO synthesis inhibitor, which suggest involvement of L-arginine-derived NO synthesis [23]. The present results support that MA and conductive heat-induced NO generation/release may be through L-arginine-derived NO synthesis, since cumulative NO that mainly comes from bacteria-mediated nonenzymatic NO generation was removed by the 1st biocapture. Elevation of NO in acupoints could be achieved through activation of endothelial and/or neuronal NO synthase. In addition, these treatments-induced de novo NO generation/release predominantly over acupoints agree with previous studies reporting that tissue NO synthase level is higher in acupoints [19–21] and suggest that acupoints exist in higher levels of L-arginine-derived NO synthesis and nonenzymatic NO generation.

Several reports have demonstrated that MA and heating cause multiple biological responses in both animals and humans [7–10, 38, 39]. These responses can occur locally at or close to the site of the stimulation, or at a distance that is mediated mainly by the neuroendocrine system. In addition, acupuncture stimulation of the acupoint or the areas located on the ground of the pressure pain sensitive site (Ashi-point) has been often used for treatment of pain-related syndromes and soft-tissue damage [10, 11, 13]. Acupuncture essentially improves local circulation and allows for a flush of analgesic or sensitizing substances, leading to pain relief [38, 40–42]. Acupuncture-like stimulation affects regional blood flow in skin, muscle, and various organs [43] and produces reflex responses of various visceral functions [44]. Our results from MA and conductive heat studies consistently suggest that NO release at acupoints is induced by the therapies. Enhanced NO improves local microcirculation and removes pathological sensitizing substances. All of these contribute to biochemical/physiological improvement and beneficial effects of the therapies.

In summary, our findings from consecutive biocaptures suggest that the NO levels biocaptured during the 1st interval over PC and LU skin regions are almost twofold higher compared to the subsequent biocapture. Conductive heat and MA with low force/rate-like reinforcement method produce elevations of both accumulative and de novo NO release over local skin regions with a high level at acupoints, which enrich blood vessels and neuronal components that may contribute to NO generation. In conclusion, these reinforcement methods induce an elevation of vasodilator (NO) release over skin regions predominantly at acupoints, and elevated NO improves local circulation and allows for a flush of analgesic or sensitizing substances, which contribute to local warmness and beneficial effects of the therapies.

## Figures and Tables

**Table 1:** Characteristics of participants.

Characteristics	Participants, *n* = 25
Women, number	14
Men, number	11
Age, mean (SEM), y	38.92 ± 3.29

Body mass index, mean (SEM)	25.98 ± 1.34
Weight, mean (SEM), pounds	162.58 ± 9.30

Asian	11
Native Hawaiian/other Pacific Islander	3
Black/African American	3
White	8

## References

[1] Manheimer E., White A., Berman B., Forys K., Ernst E. (2005). Meta-analysis: acupuncture for low back pain. *Annals of Internal Medicine*.

[2] Vickers A. J., Cronin A. M., Maschino A. C. (2012). Acupuncture for chronic pain: individual patient data meta-analysis. *Archives of Internal Medicine*.

[3] Haake M., Müller H.-H., Schade-Brittinger C. (2007). German Acupuncture Trials (GERAC) for chronic low back pain: randomized, multicenter, blinded, parallel-group trial with 3 groups. *Archives of Internal Medicine*.

[4] Biro S., Masuda A., Kihara T. (2003). Clinical implications of thermal therapy in lifestyle-related diseases. *Exp Biol Med (Maywood)*.

[5] Xi L., Tekin D., Bhargava P., Kukreja R. C. (2001). Whole body hyperthermia and preconditioning of the heart: Basic concepts, complexity, and potential mechanisms. *International Journal of Hyperthermia*.

[6] Lin C.-C., Liu X.-M., Peyton K. (2008). Far infrared therapy inhibits vascular endothelial inflammation via the induction of heme oxygenase-1. *Arteriosclerosis, Thrombosis, and Vascular Biology*.

[7] Pei W. F., Xu G. S., Sun Y., Zhu S. L., Zhang D. Q. (2000). Protective effect of electroacupuncture and moxibustion on gastric mucosal damage and its relation with nitric oxide in rats. *World Journal of Gastroenterology*.

[8] Dayanc B. E., Beachy S. H., Ostberg J. R., Repasky E. A. (2008). Dissecting the role of hyperthermia in natural killer cell mediated anti-tumor responses. *International Journal of Hyperthermia*.

[9] Kokura S., Adachi S., Manabe E. (2007). Whole body hyperthermia improves obesity-induced insulin resistance in diabetic mice. *International Journal of Hyperthermia*.

[10] Novey D. W. (2000). *Clinician's Complete Reference to Complementary & Alternative Medicine*.

[11] Beijing College of Traditional Chinese Medicine, Shanghai College of Traditional Chinese Medicine, Nanjing College of Traditional Chinese Medicine &amp; The Acupuncture Institute of the Academy of Traditional Chinese Medicine. Essentials of Chinese Acupuncture. Beijing, China: Foreign Languages Press 1980

[12] Zhang Y. Y., Liu Q. G., Xu M. (2014). Effects of twirling-rotating reinforcing and reducing technique for left ventricular morphology, concentration of ET-1 and expression of type I, III collagen mRNA in spontaneous hypertensive rats. *China Acupuncture-Moxibustion*.

[13] Chan S. H. H. (1984). What is being stimulated in acupuncture: Evaluation of the existence of a specific substrate. *Neuroscience and Biobehavioral Reviews*.

[14] Gunn CC., Ditchburn FG., King MH. (1976). Acupuncture loci: a proposal for their classification according to their relationship to known neural structures. *Am J Chin Med (Gard City N Y*.

[15] Monteiro-Riviere N. A., Hwang Y. C., Stromberg M. W. (1981). Light microscopic morphology of low resistance skin points in the guinea pig. *The American Journal of Chinese Medicine*.

[16] Moncada S., Higgs E. A. (1991). Endogenous nitric oxide: physiology, pathology and clinical relevance. *European Journal of Clinical Investigation*.

[17] Salter M., Knowles R. G., Moncada S. (1991). Widespread tissue distribution, species distribution and changes in activity of Ca2+-dependent and Ca2+-independent nitric oxide synthases. *FEBS Letters*.

[18] Dippel E., Mayer B., Schönfelder G., Czarnetzki B. M., Paus R. (1994). Distribution of constitutive nitric oxide synthase immunoreactivity and NADPH-diaphorase activity in murine telogen and anagen skin. *Journal of Investigative Dermatology*.

[19] Ma S. X. (2003). Enhanced nitric oxide concentrations and expression of nitric oxide synthase in acupuncture points/meridians. *The Journal of Alternative and Complementary Medicine*.

[20] Abraham T. S., Chen M.-L., Ma S.-X. (2011). TRPV1 expression in acupuncture points: Response to electroacupuncture stimulation. *Journal of Chemical Neuroanatomy*.

[21] Ma S. X. (2008). Biochemical physiology of nitric oxide over acupuncture points and meridians: new approach and perspectives. *Acupuncture Research*.

[22] Jou N. T., Ma S. X. (2009). Responses of nitric oxide-cGMP release in acupuncture point to electroacupuncture in human skin in vivo using dermal microdialysis. *Microcirculation*.

[23] Kimura K., Takeuchi H., Yuri K., Wakayama I. (2013). Effects of nitric oxide synthase inhibition on cutaneous vasodilation in response to acupuncture stimulation in humans. *Acupuncture in Medicine*.

[24] Ikeda Y., Biro S., Kamogawa Y. (2001). Repeated thermal therapy upregulates arterial endothelial nitric oxide synthase expression in Syrian golden hamsters. *Japanese Circulation Journal*.

[25] Ikeda Y., Biro S., Kamogawa Y. (2005). Repeated sauna therapy increases arterial endothelial nitric oxide synthase expression and nitric oxide production in cardiomyopathic hamsters. *Circulation Journal*.

[26] Li S., Chen K., Wu Y. (2003). Effects of warm needling at zusanli (ST 36) on NO and IL-2 levels in the middle-aged and old people. *Journal of Traditional Chinese Medicine*.

[27] Ignarro L. J., Fukuto J. M., Griscavage J. M., et al. (1993). Oxidation of nitric oxide in aqueous solution to nitrite but not nitrate: comparison with enzymatically formed nitric oxide from L-arginine. *Proceedings of the National Academy of Sciences of the United States of America*.

[28] Ignarro L. J. (1990). Biosynthesis and metabolism of endothelium-derived nitric oxide. *Annual Review of Pharmacology and Toxicology*.

[29] Akaike T., Yoshida M., Miyamoto Y. (1993). Antagonistic action of imidazolineoxyl N-oxides against endothelium-derived relaxing factor/•NO through a radical reaction. *Biochemistry*.

[30] Yoshida M., Akaike T., Wada Y. (1994). Therapeutic Effects of Imidazolineoxyl N-Oxide Against Endotoxin Shock through Its Direct Nitric Oxide-Scavenging Activity. *Biochemical and Biophysical Research Communications*.

[31] Ma S.-X., Li X.-Y., Sakurai T., Pandjaitan M. (2007). Evidence of enhanced non-enzymatic generation of nitric oxide on the skin surface of acupuncture points: An innovative approach in humans. *Nitric Oxide - Biology and Chemistry*.

[32] Ma S. X., Mayer E., Lee P. (2015). Transcutaneous electrical nerve stimulation increased nitric oxide-cyclic cGMP release biocaptured over skin surface of the pericardium meridian and acupuncture points in humans. *Acup & Electro-Therapeutics Res INT J*.

[33] Ma S. X., Lee P., Li X. Y., Jiang I., Ma E., Hu J. (2015). Influence of age, gender, and race on nitric oxide release over acupuncture points-meridians. *Scientific Reports*.

[34] Ha Y., Kim M., Nah J., Suh M., Lee Y. (2012). Measurements of location-dependent nitric oxide levels on skin surface in relation to acupuncture point. *Evidence-Based Complementary and Alternative Medicine*.

[35] Zhu Z. X. (1988). The advances and prospect in physiological and biophysical approaches of acupuncture meridian system. *Acupuncture Research*.

[36] Shastry S., Minson C. T., Wilson S. A., et al. (2000). Effects of atropine and L-NAME on cutaneous blood flow during body heating in humans. *Journal of Applied Physiology*.

[37] Li Y. M. (2013). Puzzles and hypotheses of acupuncture. *Chin J Integr Tradit West Med*.

[38] Kellogg D. L., Crandall C. G., Liu Y., et al. (1998). Nitric oxide and cutaneous active vasodilation during heat stress in humans. *Journal of Applied Physiology*.

[39] Sandberg M., Lundeberg T., Lindberg L., Gerdle B. (2003). Effects of acupuncture on skin and muscle blood flow in healthy subjects. *European Journal of Applied Physiology*.

[40] Tsuchiya M., Sato E. F., Inoue M., Asada A. (2007). Acupuncture enhances generation of nitric oxide and increases local circulation. *Anesthesia and Analgesia*.

[41] Sandberg M., Lindberg L., Gerdle B. (2004). Peripheral effects of needle stimulation (acupuncture) on skin and muscle blood flow in fibromyalgia. *European Journal of Pain*.

[42] Sandberg M., Larsson B., Lindberg L.-G., Gerdle B. (2005). Different patterns of blood flow response in the trapezius muscle following needle stimulation (acupuncture) between healthy subjects and patients with fibromyalgia and work-related trapezius myalgia. *European Journal of Pain*.

[43] Uchida S., Hotta H. (2008). Acupuncture affects regional blood flow in various organs. *Evidence-Based Complementary and Alternative Medicine*.

[44] Sato A., Sato Y., Suzuki A., Uchida S. (1993). Neural mechanisms of the reflex inhibition and excitation of gastric motility elicited by acupuncture-like stimulation in anesthetized rats. *Neuroscience Research*.

